# Synergistic Apoptotic Effect of Crocin and Paclitaxel or Crocin and Radiation on MCF-7 Cells, a Type of Breast Cancer Cell Line

**DOI:** 10.1155/2015/139349

**Published:** 2015-11-26

**Authors:** Faeze Vali, Vahid Changizi, Majid Safa

**Affiliations:** ^1^Technology of Radiology and Radiotherapy Department, Allied Medical Sciences School, Tehran University of Medical Sciences, No. 22, Keshavarz Boulevard, Tehran 14177-44361, Iran; ^2^Allied Medical Sciences School, Iran University of Medical Sciences, Tehran, Iran

## Abstract

*Background.* Chemotherapy, radiotherapy, and surgery are routine treatments of breast cancer. However, these methods could only improve the living survival. Nowadays the combined therapy including herbals such as crocin is to study for improving breast cancer treatment. The purpose of this study was to evaluate the effects of crocin, paclitaxel, and radiation on MCF-7 cell.* Methods.* To evaluate the effect of crocin, paclitaxel, and radiation on survival rate of MCF-7 cells MTT assay was done. To investigate the apoptotic effect of experimental groups PI-flow cytometry was used and expression of apoptotic proteins (caspase-7, caspase-9, PARP, and p53) was studied by western blot.* Results.* This study revealed that the combined therapy of 0.01*µ*mol/mL paclitaxel and 2.5 mg/mL crocin after 48 h could cause IC50 for MCF-7 cell line. This study showed that the combined therapy of 2 Gy gamma radiation with crocin could rise apoptosis in MCF-7 cell line from 21% (related to using 2 Gy gamma radiation alone) to 46.6%.* Conclusion.* Crocin and paclitaxel and crocin and gamma radiation had synergistic effect on MCF-7 cell line to get more significant apoptosis.

## 1. Introduction

Breast cancer is the most common malignant neoplasm among women in the world [1]. Due to globally uncontrolled growth in recent years, it is very important. In United States one of every eight women is facing this disease. Only in 2013 about 230,000 women and 2000 men were also added to this cancer society [2]. Although the incidence of cancer in Asia is low, its death toll is more than that in western countries. In Iran, breast cancer has the highest incidence among malignancies in women. It has been found that the incidence of breast cancer in Iran is less than that in developed countries. However, this disease is the most common cancer among the Iranian women. The incidence of this malignancy has increased in the past two decades [3].

Radiotherapy, chemotherapy, and surgery are the common treatment methods for breast cancer. Mostly surgery could not remove the tumor completely. Radiotherapy and chemotherapy could damage healthy tissues too, so that for considering healthy tissues tolerance radiation and drug doses have to be kept at a limited level. Therefore, only the living survival could be improved for a few years.

Saffron has various pharmacological effects. It could be used to cure vomiting, dental and gingival pain, insomnia, depression, seizures, cognitive disorders, diaphoretic, eupeptic, expectorant, and aphrodisiac and in the treatment of hepatic disorders, flatulence, spasm, asthma, coughs, bronchitis, colds, fever, cardiovascular disorders, cancer, and many other diseases. Crocin is the carotenoid pigments creating the redness of saffron [4]. Some studies reported the anticancer effects of crocin [5–7]. Crocin is a phytochemical to kill cancer cells without damaging the normal cells. Therefore, it could be used in combination with chemotherapy drugs to reduce the toxic effects of drugs [8].

Paclitaxel is a kind of the chemotherapy drugs. Because of paclitaxel's ability to bind to the beta tubulin subunit, it increases the production of microtubules and prevents them from depolymerization. This event consequently disrupts the cell mitosis and stops cells in the G2 phase leading to apoptosis [9]. The most important adverse effect and dose-limiting prescription of paclitaxel is the deactivation of the hematopoietic system. This is manifested by reduction of neutrophils (neutropenia), reduction of leukocytes (leukopenia), and anemia [10]. Paclitaxel may cause some cardiovascular complications such as low blood pressure (hypotension), heart rate (bradycardia), and high blood pressure (hypertension) [11]. Nausea and vomiting are the other side effects of paclitaxel [12, 13].

DNA is a critical target for ionizing radiation since it includes information to encode biomolecules, so that its damage such as single strand break (SSB), double strand break (DSB), and dimerization could threat the cell viability strongly [14, 15].

Therefore, the ionizing radiations have potential to induce DNA damage and apoptosis [16].

The term of apoptosis was first established by Kerr in 1972 to describe the physiological cell death based on morphological changes and to make differentiation from necrosis [17]. Apoptosis is a common type of cell death in eukaryotes. This process is performed during embryonic stage and tumor suppression [18].

According to increasing prevalence of breast cancer and the adverse effects of treatment programs including resistance to chemotherapy drug and damage to healthy tissues during chemotherapy and radiotherapy, this study aimed to asses synergistic apoptotic effect of crocin and paclitaxel and crocin and gamma radiation, respectively, on MCF-7 cell line.

## 2. Materials and Methods

### 2.1. Cell Lines and Reagents

MCF-7 cell line, noninvasive estrogen receptor (ER) positive, was prepared from the Pasteur Institute (Tehran, IRAN). Trypsin, crocin, 3-(4,5-dimethylthiazol-2-yl)-2, 5-diphenyltetrazolium (MTT), and propidium iodide (PI) were purchased from Sigma (Germany). Dulbecco's Modified Eagle's Medium (DMEM) high glucose with 5% fetal bovine serum was purchased from Gibco (USA). Paclitaxel was purchased from Sobhan Darou (Tehran, Iran). Rabbit monoclonal antibody of caspase-7, caspase-9, and PARP and mouse monoclonal antibody of p53 were prepared from Cell Signaling (USA).

### 2.2. Cell Culture and Research Methods

MCF-7 cells were cultured in the DMEM high glucose medium with 10% heat-inactivated fetal bovine serum, 100 units/mL penicillin, and 100 mg/mL streptomycin and maintained in a humidified atmosphere at 37°C and 5% CO_2_. Cells in the logarithmic growth period were selected for experimental studies.

### 2.3. Measurement of the Survival Rates of MCF-7 Cells with MTT Method

25000 cells were seeded in the 24-well plate with 1 mL of culture medium for 24 h. Different concentrations of 1.5 mg/mL, 2.5 mg/mL, 3.5 mg/mL, 4.5 mg/mL, and 6 mg/mL for the crocin and 0.01 *μ*M/mL, 0.03 *μ*M/mL, 0.05 *μ*M/mL, 0.1 *μ*M/mL, 0.5 *μ*M/mL, and 1 *μ*M/mL for the paclitaxel were examined separately to get IC50 (inhibition concentration) for MCF-7 at the minimum possible time. On this base the incubation times for crocin were used 24 h, 48 h, and 72 h and for paclitaxel 48 h. Then the medium was being removed and cells were incubated with 100 *μ*L (5 mg/mL dissolved in PBS) MTT and 900 *μ*L medium culture at 37°C for 4 h. For each well the supernatant was discarded, 500 *μ*L DMSO was added, and the mixture was suspended. The light absorbance (*A*) was measured at 570 nm wavelength using ELISA. Finally survival rate calculated as follows:

(1) survival  rate  of  tumor  cells  %=experimental  group  A  valuecontrol  group  A  value×100%.

All above steps were done for crocin and paclitaxel separately. As a result concentrations of 2.5 mg/mL for crocin and 0.01 *μ*M/mL for doxorubicin were selected as the optimized concentrations.

Then four groups of MCF-7 were selected to study three methods of treatments as follows: control group, second group irradiated by 2 Gy gamma radiation (Cobalt 60 source), the third group which received 2.5 mg/mL crocin with 0.01 *μ*M/mL paclitaxel, and finally the fourth group which was studied by 2.5 mg/mL crocin with 2 Gy gamma radiation for the combined group; meanwhile, we had several control groups.

### 2.4. Determination of Cell Apoptosis by Flow Cytometry

Apoptotic cells were revealed by the flow cytometry using PI staining to detect the so-called sub-G1 peak [19]. For this assay MCF-7 cells were cultured in a 6-well plate (70000 cells per well) and treated with 2.5 mg/mL crocin for 24 h, 48 h, and 72 h. The second group of cells was treated with 0.01 *μ*M/mL of paclitaxel and the third group was irradiated with 2 Gy gamma. To evaluate the combined therapy, the fourth group was treated by 2.5 mg/mL crocin and 0.01 *μ*M/mL paclitaxel for 48 h and the fifth group was treated by 2 Gy gamma and 2.5 mg/mL crocin with 24 h incubation. Then for all groups beside the control group the flow cytometry analysis was being done.

### 2.5. Western Blot to Detect the Expression of Caspase-7 and Caspase-9 and p53 and PARP of MCF-7 Cells

MCF-7 cell lines were classified and treated similar to the two previous methods. Then treated cells were detached by trypsinization, washed with PBS, centrifuged in 4000 rpm for 5 minutes, and added to RIPA (lysis buffer). In the next step all samples were put in ice for 30 minutes and vortexed every 5 minutes until those would have been homogenized well. Then samples were centrifuged 13000 rpm for 20 minutes at 4°C. The supernatant was taken out and the concentration of protein was being measured by Bradford method. Protein samples were divided into smaller amounts and kept at −80°C.

To do western blot at first running buffer and transfer buffer were prepared and then three steps were being followed up: (a) proteins were separated according to molecular weight by gel electrophoresis and (b) transferred to nitrocellulose membrane and (c) the desired protein was specified with the primary antibody and shown with the secondary antibody. Finally antibody bonds appeared with ECL on the film. The thickness of each bond was directly related to the amount of protein.

## 3. Results

### 3.1. Changes of Survival Rates of MCF-7 Cells

**Figure 1:**
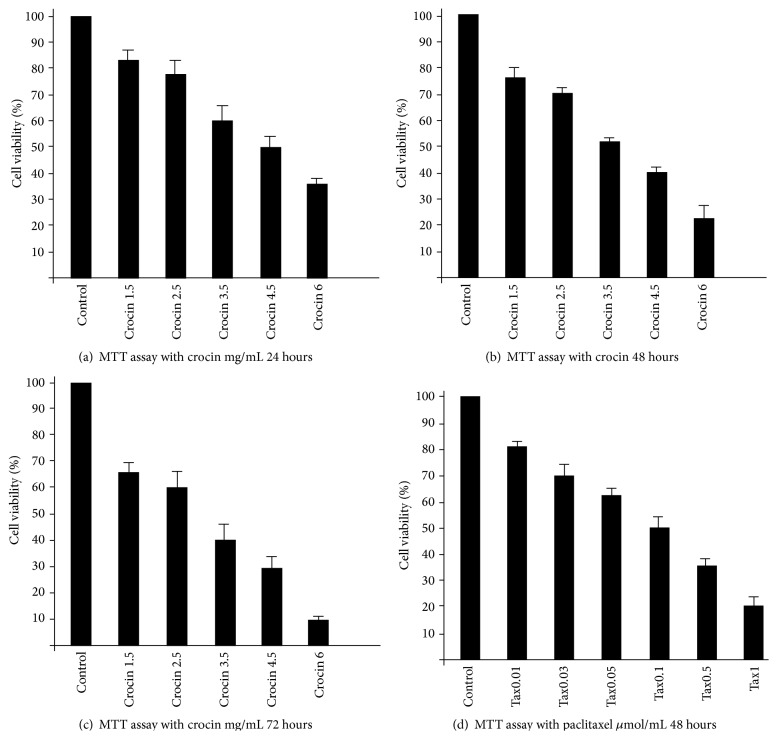
Survival rate of Mcf-7 cells with different concentration of crocin in (a) 24 h, (b) 48 h, and (c) 72 h. (d) The survival rate of Mcf-7 cells with different concentration of paclitaxel in 48 h measured by MTT.

MTT showed crocin decreased cell viability of MCF-7 cell line with increase of dose and time. IC50 for this cell line was obtained with 3.5 mg/mL crocin after 48 h treatment (*p* < 0.05) (Figure 1). IC50 was measured for treatment with 0.1 *μ*m/mL paclitaxel after 48 hours (*p* < 0.05) (Figure 1).

**Figure 2:**
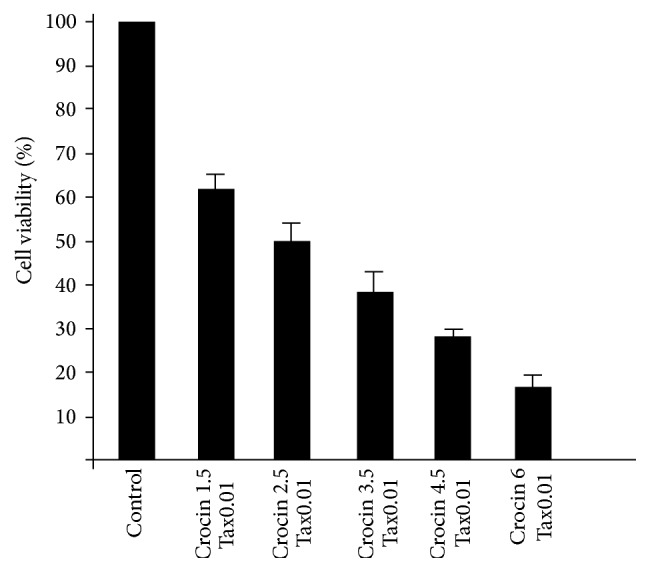
Survival rate of Mcf-7 cells with different concentration of crocin 2.5 mg/mL and 0.01 *μ*m/mL of paclitaxel 48 h.

**Figure 3:**
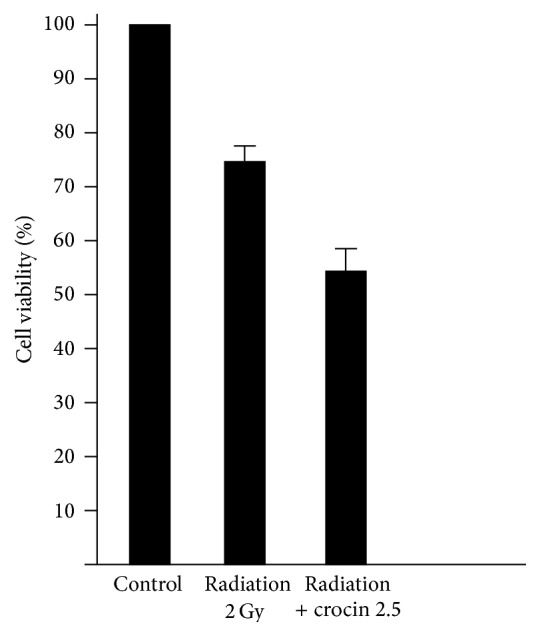
Survival rate of Mcf-7 cells with 2 Gy radiation 24 h and combination of 2 Gy radiation 24 h and crocin 2.5 mg/mL 48 h.

This study revealed that the combined therapy of 0.01 *μ*mol/mL paclitaxel and 2.5 mg/mL crocin after 48 h could cause IC50 for MCF-7 cell line (*p* < 0.05) (Figure 2). This result was found by MTT assay. Also the combined therapy of 2 Gy gamma radiation with 2.5 mg/mL crocin revealed 50% cell death (*p* < 0.05) (Figure 3). That was greater than 22% cell death for using 2 Gy radiations alone.

### 3.2. Apoptosis Changes of MCF-7 Cells

Flow cytometry with PI is used to assess apoptosis. One of the main characteristics of apoptotic cells is DNA fragmentation. Therefore, nuclear DNA content of the cells is less than that in normal cells. Nuclear-containing could be evaluated and specified in hypodiploid cells by DNA binding fluorochromes such as PI [20]. Apoptotic cell population is defined by SUBG1 peak.

**Figure 4:**
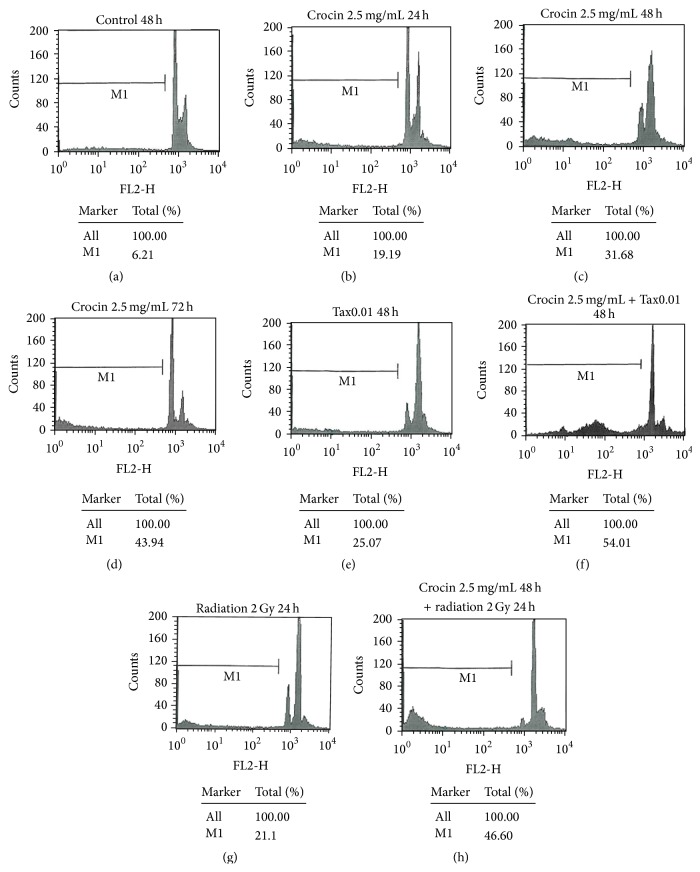
Measurement of apoptosis by PI flow cytometry: (a) control 48 h, (b) crocin 2.5 mg/mL 24 h, (c) crocin 2.5 mg/mL 48 h, (d) crocin 2.5 mg/mL 72 h, (e) paclitaxel 0.01 *μ*m/mL, (f) crocin 2.5 mg/mL + paclitaxel 0.01 *μ*m/mL, (g) radiation 2 Gy 24 h, and (h) crocin 2.5 mg/mL 48 h + radiation 2 Gy 24 h.

This study revealed that treatment with 2.5 mg/mL crocin in 24, 48, and 72 hours could cause increase of apoptosis in MCF-7 cell line with time (Figure 4). Also it was found that the combined therapy of 2.5 mg/mL crocin and 0.01 *μ*m/mL paclitaxel could increase apoptosis significantly more than that in the single therapy (*p* < 0.05) (Figure 4).

This study showed that the combined therapy of 2 Gy gamma radiation with crocin could raise apoptosis in MCF-7 cell line from 21% (related to using 2 Gy gamma radiation alone) to 46.6% (*p* < 0.05) (Figure 4).

### 3.3. The Expression of Caspase-7 and Caspase-9, p53, and PARP of MCF-7 Cells by Western Blot

**Figure 5:**
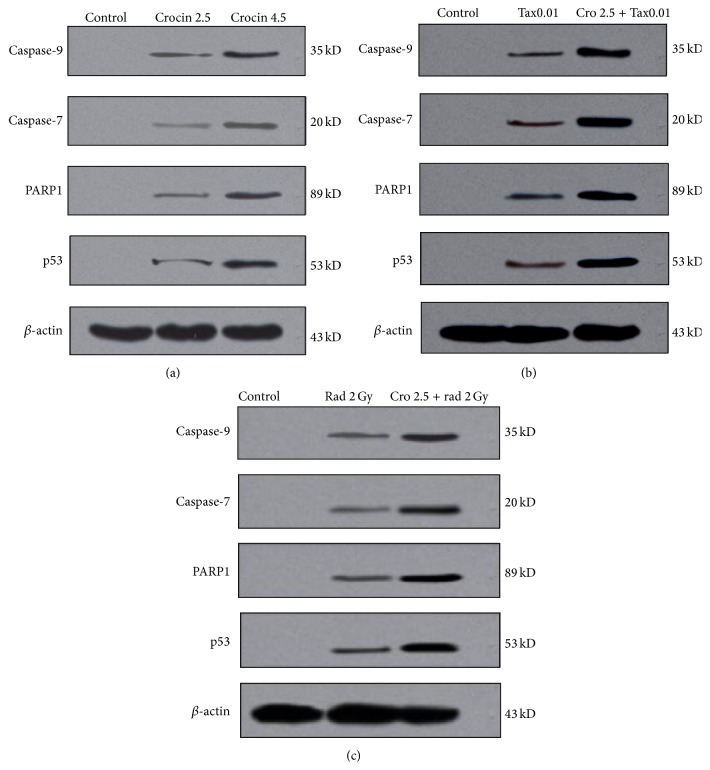
(a) Western blot of cells treated by 2.5 and 4.5 mg/mL of crocin, (b) western blot of cells treated by paclitaxel 0.01 *μ*m/mL and paclitaxel 0.01 *μ*m/mL + crocin 2.5 mg/mL, and (c) western blot of 2 Gy gamma radiation and 2 Gy + crocin 2.5 mg/mL.

Western blot test showed combined therapy of MCF-7 cell line with crocin and paclitaxel or crocin and radiation could increase the expression of 4 proteins including caspase-7 and caspase-9, p53, and PARP more than that in single therapy (*p* < 0.05) (Figure 5).

## 4. Discussion

As mentioned earlier, breast cancer is the most common malignant neoplasm among women in the world. Despite using the routine therapeutic approaches including surgery, chemotherapy, and radiotherapy in patients with cancer, their mortality remains high. It means that those therapeutic modalities need to be modified. In addition, the destructive effects of chemotherapy and radiation on normal cells are other important concerns [11]. Nowadays trends have been focused on natural products such as saffron with anticancer properties to get the better treatment for cancer.

Chryssanthi et al. showed that constituents of saffron (crocin, crocetin, and safranal) could stop the proliferation of MCF-7 cell line [12]. Our study approved this antiproliferative effect of crocin. Other scientists have approved this antiproliferative effect of crocin, saffron carotenoids pigment on HEPG2 cell, and colorectal cancer cells with no harmful effect on normal cells too [13–15]. Our study evaluated survival rate of MCF-7 cells in treatment with paclitaxel, crocin, radiation and used MTT assay. Our research showed that crocin could inhibit the proliferation of MCF-7 cells in a dose and time dependent manner. For example, when the concentration of crocin increased from 1.5 mg/mL to 6 mg/mL in 48 h, the survival rate of MCF-7 cells reduced from 75% to 23% and the survival rate of MCF-7 cells reduced from 80% to 17% in treatment of cells with paclitaxel from 0.01 *μ*M/mL to 1 *μ*M/mL in 48 h. Paclitaxel reduced the survival rate in a dose dependent manner. Similar to our results Hoshyar et al. showed dose dependent and time dependent effect of crocin.

Sun et al. revealed the apoptotic effect of crocin on Tca8113 [16]. Our study approved this effect on MCF-7 cell line. Li et al. revealed the synergistic effect of crocin and cisplatin on OS732, MG63 cells treatment. They showed the combination of crocin and cisplatin to have the strong effect of killing and suppressing of invasion cells, and it could be even more due to the expression of caspases-8 and -3 [17]. The mechanism of apoptosis is complex and based on a cascade of molecular events. It depends on energy. There are generally 2 pathways to induce apoptosis: extrinsic pathway and intrinsic pathway. These two routes are interrelated. Also T lymphocyte induces cytotoxic activity on the target cells [18]. It could be known as the third way. Caspases cut the aspartate units. This process activates the procaspases. Caspases in cascade activation could activate another caspase through a chain reaction strengthening and intensifying the route of apoptosis and rapid cell death. These enzymes are in three groups: initiator (caspases-2, -8, -9, and -10), broker (caspases-3, -6, and -7), and inflammatory (caspases-1, -4, and -5) [19–21].

PUMA gene is directly induced by P53 in response to DNA injury [22]. Expression of PUMA causes the release of cytochrome c of mitochondria stimulating cell death [23, 24]. In the inner or mitochondrial pathway of apoptosis, interaction of cytochrome c, Apaf-1, and procaspase-9 creates apoptosome [25]. Apoptosome activates caspase-9 activating executive caspases such as 3, 6, and 7 [26].

Cellular stresses such as DNA damage, radiation, or chemical carcinogens activate p53. According to the type and severity of toxicity, p53 could cause cell cycle arrest or cell death due to apoptosis. The former allows DNA repair and the latter causes cell loss [27, 28].

PARP as the cellular protein is cleaved specifically in apoptosis. Particular proteolysis of PARP happens in the DNA binding domain. Caspase-3 and caspase-7 are the most effective proteases for PARP cleavage. As a result, PARP splits into two 89 and 24 kDa subunits. These subunits are indicative of apoptosis. Therefore, in this study PI flow cytometry was used to evaluate MCF-7 cell line apoptosis and western blot was used to study the expression of proteins involved in apoptosis [29].

Saunders et al. demonstrated apoptosis in MCF-7 cells using 0–20 ng/mL paclitaxel. Our study evaluated the combined therapy of paclitaxel-crocin and paclitaxel-radiation for MCF-7 cell line and found the synergistic effect of those methods. So that 2.5 mg/mL crocin and 0.01 *μ*M/mL paclitaxel for 48 hours could cause 54.01% of cells in subG1 phase. This value for the combined therapy of 2.5 mg/mL crocin and 2 Gy gamma radiation was obtained 46.6%. The results showed that the expression of apoptotic proteins in the combination groups was significantly higher than the single groups [30].

## 5. Conclusion

The combined therapy of crocin and paclitaxel or crocin and gamma radiation has synergistic effect for increasing apoptosis and survival rate reduction of MCF-7 cell line. Therefore, it could be suggested to do more study for new breast cancer treatment. In this combined therapy concentration of crocin, drug, and treatment duration are the important factors to get higher apoptosis and lower survival rate in MCF-7 cell line.
